# Editorial: Comparative Genomics and Functional Genomics Analyses in Plants

**DOI:** 10.3389/fgene.2021.687966

**Published:** 2021-05-11

**Authors:** Xiaoming Song, Zhiyong Liu, Hongjian Wan, Wei Chen, Rong Zhou, Weike Duan

**Affiliations:** ^1^Center for Genomics and Bio-Computing, School of Life Sciences, North China University of Science and Technology, Tangshan, China; ^2^College of Horticulture, Shenyang Agricultural University, Shenyang, China; ^3^Institute of Vegetables, Zhejiang Academy of Agricultural Sciences, Hangzhou, China; ^4^Department of Food Science, Aarhus University, Aarhus, Denmark; ^5^College of Life Sciences and Food Engineering, Huaiyin Institute of Technology, Huai'an, China

**Keywords:** plant evolution, genome sequencing, molecular marker development, database construction, gene family

## Introduction

With rapidly developing sequencing technology and lower sequencing costs, over 500 plant genomes have been sequenced since the first release of the *Arabidopsis thaliana* genome in 2000. Meanwhile, more and more omics datasets have been released utilizing techniques such as genome re-sequencing, pan-genomics, RNA-Seq, metabolomics, and proteomics. The release of these datasets provides us with an excellent opportunity and rich data resources to study the evolution of plant genes. In recent years, in addition to the rapid development of bioinformatics, many types of software and tools have also become available for comparative genomics analyses. However, in the face of more and more kinds of omics data, we still need more efficient, powerful, and user-friendly tools, software, pipelines, websites, and databases.

Together with the rich omics datasets and advanced bioinformatics tools and methods, we hope to make more progress in plant comparative genomics and functional genomics. Utilizing the different types of omics data will help us to better understand the evolutionary history of plants, provide resources for molecular studies on important agronomic and economic traits, and develop new gene function verification methods. Therefore, we organized this Research Topic about “Comparative Genomics and Functional Genomics Analyses in Plants.”

This Research Topic covered a broad range of comparative and functional genomic studies. Comparative genomics analyses to better understand genes or species evolution utilizing bioinformatics, including gene family analyses and plant evolutionary analyses. Functional genomics analyses to better understand plant genes or genome evolution utilizing omics datasets and molecular biology methods, such as including *de novo* genome sequencing and pan-genomic analyses, genome re-sequencing and GWAS analyses, RNA-seq and metabolomics analyses, and characterization of novel genes.

Finally, a total of 36 outstanding works was presented in the issue of this Research Topic. All of these articles were divided into five types, including 30 Original Research, 2 Brief Research Report, 2 Data Report, 1 Review, and 1 Methods. These articles covered a wide range of fields, including gene family analyses, transcriptome, *de novo* genome, chloroplast genome, and others. Here are some representative articles selected from each of these categories.

## Gene Family Analyses

Among 36 articles, 13 were involved in the gene family analyses in different species, including Apiaceae crops (ARF), Chinese cabbage (GRP), cotton (UGD and Rboh), cucumber (HSP90, BES1, and MYB), Gramineae crops (PLTs), green algae (PHT), pepper (bHLH), potato (PRXs), tobacco (SNAT), and tomato (C2H2-ZFP) (Supplementary Table 1). Most of these studies conducted comprehensive analyses of the gene family, such as gene family identification, gene structure, conserved motif, phylogenetic relationship, orthologous and paralogous genes, collinearity analysis, and expression pattern analyses. All of these studies provide rich resources for the comparative analysis of the related gene families in plants.

## Transcriptome

In the Research Topic, there were about 12 articles that belonged to the transcriptomic studies (Supplementary Table 2). Among which, most RNA-seq experiments were designed as various biotic and abiotic stresses, including cold (Huang et al.; Yang et al.), heat (Cai et al.), drought (Kim et al.), phosphate deficiency (Zhang et al.), turnip mosaic virus infection (Lyu et al.). Combining with RNA-Seq, several other technologies were also used to explore the gene expression or gene regulatory pathway. For example, Lyu et al. used the RNA-Seq and quantitative iTRAQ-LC-MS/MS to reveal the resistance mechanism against TuMV in Chinese Cabbage. Liu et al. explored the height growth-related genes by combining QTL analysis and transcriptome in *Salix matsudana*. In addition to mRNA expression analysis, some studies have also focused on the expression of non-coding RNA, such as microRNAs (Zhang et al.) and circular RNAs (Yang et al.). All of these transcriptome datasets can be downloaded using the accession number list in the Supplementary Table 2 from NCBI website (https://www.ncbi.nlm.nih.gov). These RNA-Seq datasets will lay the foundation for gene function analysis in the future.

## *DE NOVO* Genome

In this topic, four studies reported and released the plant genomes, which provided the basic and rich resources for comparative and functional genomics analysis in plants (Table 1).

**Table 1:** The list of chloroplast genome and *de novo* genome studies in this Research Topic.

**Category**	**Species**	**Database**	**Accession No**.	**Links**
Chloroplast genome	*Salvia yangii* *Salvia* *miltiorrhiza* *f. alba* *Salvia miltiorrhiza* *Salvia officinalis* *Salvia przewalskii* *Salvia japonica* *Salvia chanryoenica* *Salvia bulleyana* *Salvia rosmarinus*	GenBank	MT012421 MT012420 NC_020431 NC_038165 NC_041091 NC_035233 NC_040121 NC_041092 NC_027259	Gao et al.
	*Curcuma rosesana* *Curcuma amarissima* *Curcuma sichuanensis* *Curcuma elata* *Curcuma zanthorrhiza* *Curcuma longa* *Curcuma yunnanensis* *Curcuma sp.1* *Curcuma sp.2* *Curcuma wenyujin* *Curcuma phaeocaulis* *Curcuma aromatica* *Curcuma alismatifolia* *Curcuma flaviflora*	GenBank	MT395655 MT395645 MT395646 MT395649 MT395651 MT395644 MT395657 MT395653 MT395648 MT395650 MT395652 MT395654 MT395647 MT395656	Liang et al.
	*Alchemilla argyrophylla* *Alchemilla pedata* *Dasiphora fruticosa* *Fragaria iinumae* *Fragaria nipponica* *Fragaria pentaphylla* *Fragaria orientalis* *Fragaria chiloensis* *Fragaria virginiana* *Fragaria mandshurica* *Fragaria vesca* *Rosa multiflora* *Rosa odorata* *Hagenia abyssinica*	GenBank	MT382661 MT382662 MF683841 KC507759 KY769125 KY434061 KY769126 JN884816 JN884817 KC507760 KC507760 NC_039989 KF753637 KX008604	Rono et al.
*De novo* genome	*Gastrodia* *elata*	NCBI	PRJNA394702	Chen et al.
	*Megacarpaea delavayi*	NCBI	PRJNA630110 PRJCA002887	Yang et al.
	*Andrographis paniculata*	NCBI	PRJNA549104	Liang et al.
	*Sargassum fusiforme*	NCBI	PRJNA597239 SRR12124361	Wang et al.

Liang et al. reported a high-quality reference genome for *Andrographis paniculata* (Chuanxinlian) using the PacBio and Illumina sequencing. The assembly genome was 284 Mb, and contig N50 was 5.14 Mb. The contigs were further assembled into 24 pseudo-chromosomes using Hi-C technique. This high-quality genome builds the foundation for exploring the biosynthetic pathways of various medicinal compounds in the future.

Yang et al. reported a draft genome for *Megacarpaea delavayi* (Brassicaceae), a plant that lived in the high mountains of southwest China at high altitudes. The assembly genome was 883 Mb, and contained a total of 41,114 protein-coding genes. They found that *M. delavayi* experienced an independent whole-genome duplication (WGD), paralleling to those WGDs of Iberis, Biscutella, and Anastatica in the early Miocene. *M. delavayi* specific and fast-evolving genes were mainly involved in “DNA repair” and “response to UV-B radiation,” which might help it survive in high-altitude environments. This genome will provide valuable resources for studying the adaptation of plants to high-altitude habitats.

Chen et al. reported a genome of *Gastrodia elata*, which is an achlorophyllous orchid plant that displays a distinctive evolutionary strategy to adapt to the non-photosynthetic lifestyle. The assembly genome of *G. elata* was 1.12 Gb with a contig N50 size of 110 kb and scaffold N50 size of 1.64 Mb. The genes related to photosynthesis, leaf development, and plastid division pathways were found to be lost or under relaxed selection during the evolution. Thus, this genome provides a good resource for investigating the evolution of orchids and other achlorophyllous plants.

Wang et al. reported the first draft genome of the Seaweed *Sargassum fusiforme* using PacBio and Illumina technology. The assembled length of *S. fusiforme* genome was ~394.4 Mb with a contig N50 value of ~142.1 Kb, and a total of 20,222 putative genes were detected. This genome can be used as a genomic reference for the discovery of functional products and evolutionary studies in the Chromista kingdom.

All of these genome datasets can be downloaded using the accession number list in the Table 1 from NCBI website.

## Chloroplast Genome

In addition to the *de novo* genome, there were three studies about the chloroplast genome in this Research Topic (Table 1).

Gao et al. reported two whole chloroplast genomes of Salvia medicinal plants and compared them with seven other Salvia chloroplast genomes. Salvia species have been widely used as medicinal plants, which played important roles in the treatment and recovery of individuals with COVID-19. Gao et al. identified ten mutation hotspots as candidate DNA barcodes for Salvia species. Especially, they detected the transfer of nine large-sized chloroplast genome fragments into the mitochondrial genome.

Liang et al. reported the complete chloroplast genome of fourteen Curcuma species and constructed the phylogenetic tree of the 25 Zingiberaceae species. The 25 complete chloroplast genomes were ranging from 155,890 to 164,101 bp, and six divergent hotspots regions were detected. The phylogenetic trees showed that Musaceae was the basal group in Zingiberales, and Curcuma had a close relationship with Stahlianthu. This study provided the high-quality chloroplast genomes for Zingiberaceae study in the future.

Rono et al. reported the complete chloroplast genomes of two Alchemilla (Rosaceae) species *Alchemilla pedata* and *A. argyrophylla*. The chloroplast genomes were a typical circular quadripartite structure with the length of 152, 438 and 152,427 bp for *A. pedata* and *A. argyrophylla*, respectively. The high variation in five regions located in the intergenic spacers was detected through the comparative analysis with eight other Rosaceae species. Based on 26 chloroplast genomes of Rosaceae species, phylogenetic analysis revealed a monophyletic clustering of Alchemilla nested within subfamily Rosoideae. These complete chloroplast genomes will contribute to species delineation and further evolutionary studies in Rosaceae species.

All of these chloroplast genome datasets can be downloaded using the accession number list in the Table 1 from NCBI website.

## Author Contributions

XS compiled the contributions from all authors. All authors contributed to the article and approved the final version of the manuscript.

## Conflict of Interest

The authors declare that the research was conducted in the absence of any commercial or financial relationships that could be construed as a potential conflict of interest.

