# Supplementary material for: Editorial: Comparative Genomics and Functional Genomics Analyses in Plants
Source: Front Genet. 2021 May 11;12:687966. doi: 10.3389/fgene.2021.687966 (PMC8148215; doi:10.3389/fgene.2021.687966)
Supplement: Supplementary file 1 [file Table_1.pdf]

Table 1. The list of gene family analyses in this research topic.

**Species**	**Latin name**	**Gene family**	**Full name**	**Links**
Apiaceae crops	*Apium graveolens, Daucus carota, Coriandrum sativum*	ARF	Auxin response factors	Pei et al.
Chinese cabbage	*Brassica rapa*	GRP	Gycine-rich protein	Lu et al.
Cotton	*Gossypium hirsutum*	UGD	UDP-glucose dehydrogenases	Jia et al.
Cotton	*Gossypium barbadense*	Rboh	Respiratory Burst Oxidase Homologs	Chang et al.
Cucumber	*Cucumis sativus*	HSP90	Heat shock protein 90	Zhang et al.
Cucumber	*Cucumis sativus*	BES1	BRI1-EMS-suppressor 1	Gao et al.
Cucumber	*Cucumis sativus*	MYB	Myeloblastosis	Cheng et al.
Gramineae crops	*Brachypodium distachyon, Hordeum vulgare, Oryza rufipogon, Oryza sativa, Sorghum bicolor, Setaria italica, Zea mays*	PLTs	Polyol Transporters	Kong et al.
Green algae	*Chlamydomonas reinhardtii*	PHT	Phosphate transporter	Wang et al.
Pepper	*Capsicum annuum*	bHLH	Basic helix–loop–helix	Zhang et al.
Potato	*Solanum tuberosum*	PRXs	Class III peroxidase gene family	Yang et al.
Tobacco	*Nicotiana tabacum*	SNAT	SNAT	Zhang et al.
Tomato	*Solanum lycopersicum*	C2H2-ZFP	C2H2-type Zinc Finger	Zhao et al.
