# Supplementary material for: Editorial: Comparative Genomics and Functional Genomics Analyses in Plants
Source: Front Genet. 2021 May 11;12:687966. doi: 10.3389/fgene.2021.687966 (PMC8148215; doi:10.3389/fgene.2021.687966)
Supplement: Supplementary file 2 [file Table_2.pdf]

Table 2. The list of transcriptome studies in this research topic.

Species	Latin name	Treatment/Trait	Accession number (NCBI)	Links
Betula	*Betula luminifera*	Phosphate deficiency	GSM4105633–GSM4105640	Zhang et al.
Camphortree	*Cinnamomum camphora*	Essential oils	SRR11362617-SRR11362618<br>SRR11362613-SRR11362614<br>SRR12576811-SRR12576812	Hou et al.
Chinese cabbage	*Brassica rapa*	Bolting	PRJNA605481	Wei et al.
Chinese cabbage	*Brassica rapa*	Turnip mosaic virus	GSE151932	Lyu et al.
Corkscrew willow	*Salix matsudana*	Height growth	CNP0001576 (CNGBdb)	Liu et al.
Flax	*Linum usitatissimum*	Different tissues	PRJNA634481	Dmitriev et al.
Red-flowered Black Mangrove	*Lumnitzera littorea*	Herkogamy	SRP127706	Zhang et al.
Rice	*Oryza sativa*	Heat stress	PRJNA633211	Cai et al.
Rice<br>Arabidopsis	*Oryza sativa*<br>*Arabidopsis thaliana*	Photosynthesis	SRR10907568–SRR10907573<br>SRR10913120-SRR10913123<br>SRR10913307–SRR10913312<br>SRR10914245–SRR10914250<br>SRR10914757–SRR10914762<br>SRR10915176–SRR10915181	Shi et al.
Saccharum grass	*Saccharum spontaneum*	Cold stress	PRJNA636260; PRJNA635765	Huang et al.
Soybean	*Glycine max*	Drought stress	GSE153660	Kim et al.
Tomato	*Solanum lycopersicum*	Low temperature	SRR12489163–SRR12489168	Yang et al.
